# A Case of Complicated Labor

**Published:** 1856-11

**Authors:** Hiram Nance

**Affiliations:** Lafayette, Ill.


ARTICLE II.—*A Case of Complicated Labor.* By HIRAM NANCE, M.D. Lafayette, Ill.

I was called, on the morning of the 5th of September, to visit an Irish lady, residing four miles distant. The messenger who came for me seemed to be in a great hurry, and urged me to go with all speed. I obeyed the summons promptly, and, when within about one mile of the house, I met the husband who was coming after me to urge me on. He remarked, "Hurry on, Doctor, my wife has been delivered of one child, and is going to have another." When I arrived at the house, I found an Irishwoman officiating as midwife. I inquired how the lady was: she said, "Very bad, Doctor; very bad. I immediately pulled off my coat, and, in making an examination, found a placenta delivered with the other end of the cord attached to another child in utero. I placed a ligature around the cord, and removed the placenta. Then I examined and made diligent search for the cord that belonged to the child that was born previous to my arrival; but the search was in vain, I could find nothing of it. I interrogated the Irish midwife in regard to it. She admitted the cord to be of the ordinary length, and that she separated the child from it: but the cord could not be found. What had become of it? My only answer to this question, is, that ignorant midwives, and, shall I say, sometimes inexperienced physicians? are too eager to deliver the placenta after the birth of the child, and, now, that something presents itself that will bear *some pulling*, they pull and pull until the cord is broken off; laying the patient liable to profuse flooding and to inversion of the uterus. Though she escape both of these, the cord being broken off is a serious perplexity, and we thereby loose the guide to the placenta.

Not finding the cord of the first child, and the placenta of the second one being delivered, I examined to ascertain the presentation of the child yet to be delivered. The mouth of the womb seemed to be fully open, and, on examination, I found the head resting above the symphosis pubis; the child was entirely doubled upon itself. By the side of the head, forced down on the perineum, was the left hand and foot. Here was a peculiarly complicated presentation—head, hand, and foot all at one time, and the uterus contracting most powerfully. The contraction was not regular, as was proved by an examination of the parieties of the abdomen. The child seemed to be forced down in the lower part of the uterus, and entirely doubled upon itself, with the placenta of the first child and one foot (the right) retained in an hour-glass-like contraction at the fundus. For a more minute description of this contraction of the womb, see *Ramsbotham's Process of Parturition*, Plate XLIX. Fig. 136. I waited for a short time, examining to see what effect the pains would have upon the child in this situation. I was very soon convinced that no pains could ever effect anything favorable while the child remained in this situation. It was impossible to push up the hand and foot, and make room for the occiput; the only alternative left, was, to bring down the feet, and thereby turn the child. After giving her a large dose of opium, she became quite easy, seemed to have no pain, slept a little, and said she was "comfortable." I again introduced my hand, but found the uterus still contracting firmly. After waiting some longer, I again made an examination, and found the parts *very little* relaxed. I made diligent search for the other foot, but to no purpose, it was impossible to find it, it was certainly at the fundus of the uterus firmly enclosed in an hour-glass-like contraction. The best authorities say, "if you cannot bring down both feet in turning, bring down one." As it was impossible to get them both, I brought down the one without any trouble, as it already rested at the os externum. On making traction by this foot, the child did not seem to be loosened the least from its situation; I then made slight traction by the foot with my left hand, and introduced my right hand and raised the head from its situation, and thereby effected the turning of the child, brought it down to its head, then brought down the chin and delivered it.

Immediately the child was delivered, I again examined, and found the uterus firmly contracting, but could not find the placenta of the first child, which was still retained at the fundus, firmly held by the irregular contraction before spoken of. I waited for an hour and a half or two hours, hoping that the firm contractions of the womb would expel it, but all to no purpose. I again examined, and could feel a part of the placenta presenting at the os uteri, which was high up in the pelvis; I managed to get hold on it, and used all the traction I dared to in an attempt to remove it. By steady traction with one hand, with the other applied externally on the uterine tumor, I attempted its removal, but this means failed. I then gave her another full opiate, and introduced my hand into the womb as far as I possibly could, and was then thorougly convinced that firm adhesion had taken place, and that my best efforts for its removal would probably prove abortive. There was but little hemorrhage, as nothing seemed to relax the firm contraction. I attempted its removal by peeling it off, and did remove parts of it; but the pain necessarily following such an operation, was so great that it seemed beyond endurance. The adhesion was so firm, that it was impossible for me to distinguish between the parieties of the uterus and the placenta. After removing, as I remarked, only a small part, and finding that my patient could not endure it any longer, I desisted: gave her another opiate, and waited about seven or eight hours, and again attempted its removal, but with no better success.

I was now convinced that its removal was incompatible with life, and that the more attempts I made for its extraction, the worse it would be for my patient; and determined to trust to the powers of nature—and in those powers I reposed but little confidence.

My prognosis, I need hardly say, was unfavorable; and this opinion I expressed to the husband and friends of my patient. I visited her every day. Milk was secreted, though to a limited extent. On the 6th and 7th, she seemed quite lively; laughed and talked; took sufficient nourishment; pulse rather quick and small; surface rather cool. On the 8th and 9th, the symptoms nearly the same, connected with a profuse, watery, sanious discharge, putrescent, and very offensive to the smell. What is remarkable, she had no after-pains; the pulse became quicker and weaker; she became restless; had frequent sighing, occasional vomiting, and, finally, sank on the evening of the 10th.

There seemed to be no inflammatory symptoms present; no tenderness per vaginum, or over the hypogastric region; neither was there any swelling or unusual fulness of the bowels; she secreted water, and passed it freely; her bowels also moved quietly. She seemed to sink as if under the influence of a powerful sedative. Would a putrid mass, remaining in the uterus, cause death, as if a powerful sedative had been given? In this case it certainly acted so, and produced death, not by inflammatory symptoms but by its sedative influence.

That there was adhesion pretty nearly through the whole extent of the placenta, is evident; examination did not only prove this, but if there had not been, flooding would certainly have been more copious. It is truly astonishing to me, that hemorrhage was no worse, when the placenta of the second child was born before it.

In regard to the removal of adherent placenta, I will cite you to *Ramsbotham's System of Parturition*, page 312. He says: "Whenever half an hour or an hour has elapsed since the birth, without the appearance of any discharge, while at the same time three or four smart uterine contractions have taken place, we may begin to suspect not only that the placenta is morbidly adherent, but that through its whole extent; because, if any part were separated, some vessels must be rendered patulous."

He says, further, "That sometimes the adhesion is so strong that it is *impossible to peel it off from its attachment;* that instances are sometimes met with in which portions of the placenta are so closely attached to the uterine surface that they *cannot, by any means, be removed.*"

The Doctor goes on further to state, that he has opened more than one body where the bond of union was so firm between the uterus and the placenta, that it could not be defined.

All can see the difficulty that would be brought to bear in meeting with such a case; and I claim that my case was a case of this kind. If any physician, *under such circumstances*, can accomplish more than I have, and advise a better course, let me hear from him. My paper has already extended to greater limits than I intended; yet my essay is quite imperfect.

